# Case Report: rTMS for recurrent major depressive disorder and generalized anxiety disorder comorbid with atrial fibrillation

**DOI:** 10.3389/fpsyt.2026.1719274

**Published:** 2026-02-25

**Authors:** Vipul Reddy, Nadia Frutkin, Abeer Singh, Manya Pappu, Khimaya Raval, Mark Odron, Charles Vigilia, Kenneth Blum, David Baron, Keerthy Sunder

**Affiliations:** 1 Division of Clinical Neuromodulation Research, Karma TMS, Palm Springs, CA, United States; 2 Palm Desert High School, Palm Desert, CA, United States; 3 La Quinta High School, La Quinta, CA, United States; 4 Taft Charter High School, Woodland Hills, CA, United States; 5 Sunder Foundation, Palm Springs, CA, United States; 6 Western University Health Sciences Centers, Pomona, CA, United States; 7 Department of Psychiatry, University of Vermont, Burlington, VT, United States; 8 Institute of Psychology, Eotvos Loránd University, Budapest, Hungary; 9 Department of Psychiatry, Wright State University Boonshoft School of Medicine, Dayton, OH, United States; 10 Centre for Genomics and Applied Gene Technology, Institute of Integrative Omics and Applied Biotechnology, Nonakuri, West Bengal, India; 11 Department of Psychiatry, Riverside School of Medicine, Riverside, University of California, Riverside, Riverside, CA, United States

**Keywords:** atrial fibrillation (AF), dorsolateral prefrontal cortex (DLPFC), GAD-7 (Generalized Anxiety Disorder-7 item scale), major depressive disorder (MDD), neuromodulation, PHQ-9 (Patient Health Questionnaire-9), repetitive Transcranial Magnetic Stimulation (rTMS)

## Abstract

This paper examines the utilization of repetitive transcranial magnetic stimulation (rTMS) to treat recurrent Major Depressive Disorder (MDD) and Generalized Anxiety Disorder (GAD) in a patient comorbid with atrial fibrillation (AF), a type of common heart arrhythmia. Symptoms of AF can increase the risk of MDD and GAD, negatively impacting quality of life. Repetitive Transcranial Magnetic Stimulation (rTMS) is an FDA-approved modality for treatment-resistant major depressive disorder (MDD) and has shown promise for off-label treatment of anxiety disorders. Quantitative psychometric questionnaires were administered weekly, using the PHQ-9 (Patient Health Questionnaire-9) and GAD-7 (Generalized Anxiety Disorder-7), which revealed symptom reductions of 55% and 22.2%, respectively.

## Introduction

Atrial fibrillation (AF) is a prevalent heart disorder, identified by an irregular heart rhythm called arrhythmia. AF is characterized by rapid atrial electrical activity, leading to accelerated and irregular ventricular activity, loss of atrial mechanical function, and an increased risk of atrial thrombus formation (1). AF causes an increase in the risk of health complications such as stroke, heart failure, and overall physiological decline. AF is a highly prevalent cardiovascular disease, with it being the most common type of arrhythmia. Moreover, approximately 12 million Americans are projected to be afflicted with AF by 2030 (2, 3).

Studies looking into the prevalence of psychological disturbances in individuals with AF have revealed that some patients may have an increased risk of depression. Polikandrioti et al. assessed 170 patients afflicted with AF for depression using the Hospital Anxiety and Depression Scale (HADS), which evaluates the patient’s level of depression. The HADS assessment revealed that 15.5% of AF patients had moderate levels of depression, and 20.2% had high levels of depression (4). A similar study by Frasure-Smith et al. aimed to evaluate depressive symptoms in patients with AF using Beck’s Depression Inventory-II (BDI-II). From a pool of 974 participants, 32% had symptoms of mild to moderate depression, indicated by a BDI-II score of ≥ 14 (5). The study revealed that elevated depression scores may cause an increased risk of mortality, which is why demonstrating the importance of treating patients manifesting with depressive symptoms and heart disease is necessary.

Anxiety may also be a leading psychological comorbidity in AF patients. Polikandrioti et al. recorded that out of 170 AF patients, 26.6% of patients recorded moderate levels of anxiety, and 34.9% recorded high levels of anxiety (4). Lane et al. found that symptoms of anxiety were common among patients with atrial fibrillation. In their study, 70 patients completed anxiety questionnaires at the time of initial diagnosis, 6 months post-diagnosis, and 12 months post-diagnosis. The results showed that 38.5%, 30.9%, and 35.7% of patients, respectively, reported experiencing symptoms of anxiety at these time points (6). These findings display that anxiety may not be an incidental experience in AF patients, but a persistent psychological comorbidity that can remain months after AF diagnosis.

This study focuses on how individuals with AF have a heightened risk of developing depression symptoms that can be treated with repetitive Transcranial Magnetic Stimulation (rTMS). Repetitive transcranial magnetic stimulation (rTMS) has emerged as a promising therapeutic alternative for patients with treatment-resistant depression. rTMS, a non-invasive neuromodulation therapy, delivers magnetic pulses to stimulate specific brain regions, particularly the left dorsolateral prefrontal cortex (DLPFC), which plays a primary role in mood regulation and executive functioning. A 10 Hz (high-frequency) stimulation of the left DLPFC has been shown to increase cortical excitability and synaptic plasticity, as well as modulate brain activity associated with depression. By increasing brain-derived neurotrophic factor (BDNF) and enhancing long-term potentiation, rTMS restores and rebuilds a functional balance in underactive brain regions (7).

The most common side effect of rTMS is varying levels of pain and discomfort, with patients often experiencing mild scalp tenderness. Pain may also occur due to patient susceptibility, improper coil placement, and magnetic pulse intensity and frequency (8). Other common side effects include transient dizziness and headaches, which occur in up to 1/3 of patients (9, 10). Lastly, rTMS can potentially induce seizures as a serious side effect, but the risk is very low, estimated to be < 0.003%, or 1 in every 30,000 treatments (11, 12). In addition, rTMS has been administered in patients with a history of seizure disorders and brain lesions, despite increased seizure risk (13, 14). Screening these patients and adhering to safety guidelines minimizes the risk of seizures from rTMS.

We report the case of a 69-year-old female with recurrent MDD and GAD, who was diagnosed with chronic atrial fibrillation. She was diagnosed with post-traumatic stress disorder at age 49, following adverse childhood experiences, including sexual trauma experienced between the ages of 4 and 9. She described symptoms of distressing thoughts, hypervigilance, and an exaggerated startle response. Furthermore, she reported experiencing depressive symptoms since her childhood. She endorsed trying multiple antidepressants, with her first antidepressant trial taking place at age 26.

At the time of the presentation, the patient reported symptoms consistent with MDD, such as persistent low mood, anhedonia, fatigue, and low motivation. In addition, the patient endorsed having symptoms consistent with General Anxiety Disorder (GAD), such as experiencing excessive worry, having difficulty relaxing, and feeling on edge. The patient had undergone multiple antidepressant trials, including bupropion XL (150 mg daily for 12 weeks in 2024), which caused weight gain, and fluoxetine (20 mg daily for 8 weeks in 2024), which was ineffective. Desvenlafaxine (50 mg daily for 10 weeks in 2024) was discontinued because of worsening depressive symptoms. Mirtazapine (7.5 mg daily for 12 weeks from August to October 2024) was also ineffective. In addition, the patient took sertraline from February 2025 to April 2025 up until the beginning of her TMS treatments, but discontinued it due to side effects, including tremors, nosebleeds, and bruising. Lastly, the patient also received psychotherapy weekly from 2003 to 2013, but it was discontinued due to lack of efficacy.

The patient was diagnosed with persistent atrial fibrillation in November 2023. She then had one cardiac ablation in April 2024 and a second cardiac ablation in September 2024 after a return of symptoms. Thus, her atrial fibrillation was stable at the time of TMS treatments. Furthermore, the patient had a Watchman device procedure in March of 2025 to prevent blood clots from her non-valvular AF. While undergoing TMS treatments, the patient was taking clopidogrel following her Watchman device procedure. In addition, the patient was not taking medications specifically targeting atrial fibrillation rate or rhythm control.

## Methods and materials

### Subject

The participant was recruited based on a clinical diagnosis of MDD refractory to 2 antidepressants of different classes, as well as comorbid anxiety and atrial fibrillation. The participant did not have any contraindications to TMS treatment, including ferromagnetic implants within 12 inches of the cranium, a cardiac pacemaker, an implantable cardioverter defibrillator, or a history of epilepsy or brain lesions. The subject was informed about the mechanics of TMS, possible side effects, and the treatment protocol, including the Motor Threshold Determination procedure. In addition, the subject was informed that they can choose to stop the treatment at any time. The subject consented to Psychometric questionnaires, MT determination Procedures, and a treatment plan of 36 DASH protocol treatments to the left dorsolateral prefrontal cortex (L-DLPFC) over the course of 8 weeks.

### Repetitive transcranial magnetic stimulation treatment

**Figure 1:**
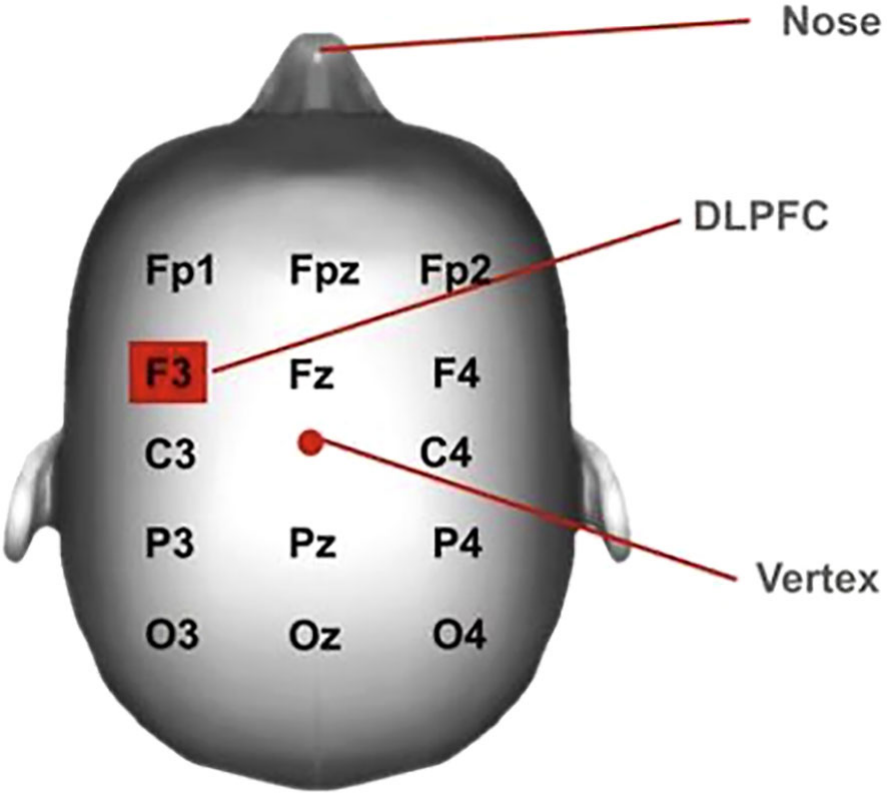
rTMS treatment location.

rTMS was administered by certified neurotechnologists using the Stimware^®^ software installed in the Apollo TMS Therapy System. Using the 10–20 system, the F3 location (Left Dorsolateral Prefrontal Cortex), as shown in **Figure 1**, corresponding to Brodmann areas (BA) 8 and 9, was approximated on the scalp (15). The motor threshold (MT) was determined at a location in the primary motor cortex that elicited a contralateral thumb twitch at the lowest intensity. Treatment parameters for left DLPFC stimulation included a frequency of 10 Hz, 3000 pulses, 40 pulses per train, 75 trains per session, and an intertrain interval of 11 seconds. The left DLPFC was approximated 5.5 cm anterior to the motor cortex, and each treatment was targeted at this location for 18 minutes and 34 seconds (16). Treatment intensity was capped at 90% of the MT, which the subject tolerated without scalp discomfort, headache, or seizure.

### Psychometric questionnaire administration

The rTMS treatment response was systematically monitored using standardized psychometric questionnaires. The Patient Health Questionnaire-9 (PHQ-9) and Generalized Anxiety Disorder-7 (GAD-7) were administered to the patient weekly throughout treatment.

## Results

To calculate the percentage change in initial and post-treatment PHQ-9 and GAD-7 scores, we used baseline versus final assessment values. PHQ-9 scores decreased from 20 to 9, a 55% decrease. In addition, GAD-7 scores decreased from 18 to 14, a 22.2% decrease.

## Discussion

**Figure 2:**
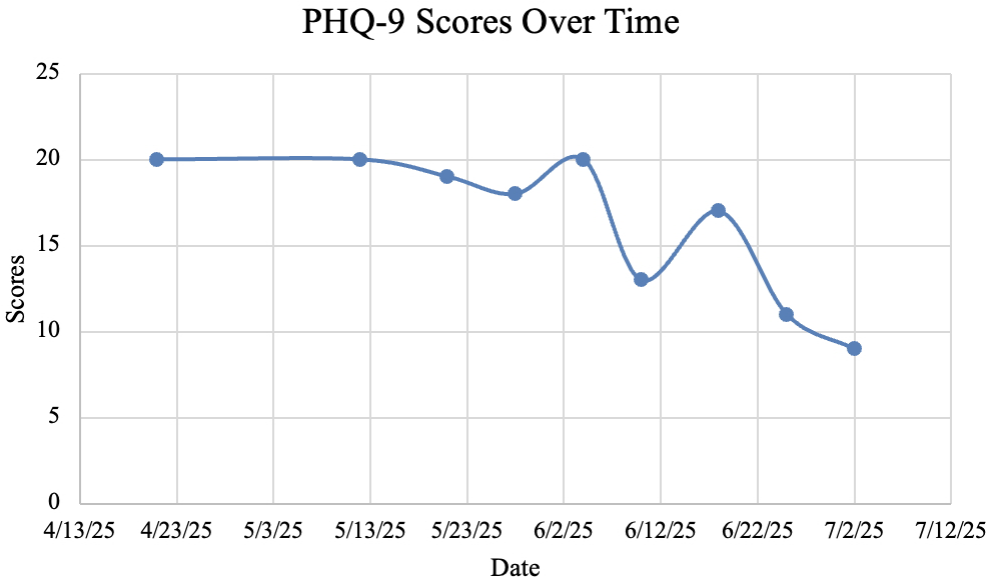
PHQ-9 scores.

**Figure 3:**
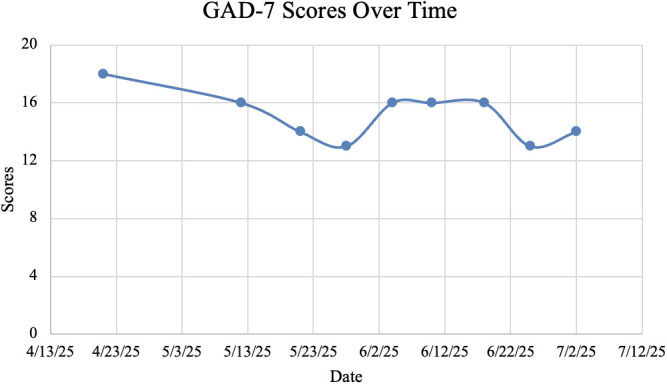
GAD-7 scores.

Our study demonstrates that rTMS was effective in treating MDD symptoms in a patient with atrial fibrillation, as evidenced by a 55% decrease in PHQ-9 scores (**Table 1**; **Figure 2**). Additionally, the patient exhibited a modest improvement in GAD symptoms, with a 22.2% decrease in GAD-7 scores (**Table 1**; **Figure 3**). The patient’s PHQ-9 scores exhibited a non-linear trajectory due to increased acute psychosocial stressors during week 7 of her treatment course, causing her PHQ-9 to rise to 17 from 13 the previous week. Following TMS sessions, the patient reported an improvement in her overall mood and found herself expressing a more positive affect. The patient also endorsed improved sleep quality, characterized by greater ease of falling asleep. Furthermore, the patient reported increased motivation, as evidenced by her willingness to leave her home more frequently. Notably, the patient also began driving a car by herself for the first time in approximately two years. Additionally, the patient gained renewed interest in watching her favorite basketball team alongside her family. Lastly, the patient remained stable throughout treatment, with no adverse cardiovascular events.

**Table 1:** PHQ-9 and GAD-7 scores by week.

Test scores	4/21/25	5/12/25	5/21/25	5/28/25	6/4/25	6/10/25	6/18/25	6/25/25	7/2/25
PHQ-9	20	20	19	18	20	13	17	11	9
GAD-7	18	16	14	13	16	16	16	13	14

Recognizing and treating depression in AF patients is a vital component in improving the quality of life for AF patients. Dabrowski et al. found that patients with various types of AF had a high prevalence of depressive symptoms and experienced significantly reduced quality of life. 28.6-35.9% of patients reported disruptions in work life, 23.8-33.9% reported disruptions in sex life, and 10.3-21.4% reported disruptions in family life. Overall, reduced quality of life was evident in their diminished emotional well-being, tendency to socially isolate, lower energy levels, and reduced interest in activities (17).

From a clinical perspective, one important factor to consider in patients with depression comorbid with atrial fibrillation is the possibility of medication non-adherence. In a study by Reading et al., it was found that 191 out of 2,500 (7.64%) patients with atrial fibrillation and depression self-reported non-adherence to prescription medication (18). Comorbidity increases the number of medications a patient must take, adding a burden of complicated regimens and increasing the likelihood of side effects. Thus, our study demonstrates that TMS can be an effective option for reducing the burden of polypharmacy in patients managing multiple conditions.

To the best of our knowledge, rTMS is understudied in patients with cardiac comorbidities. Limited studies have been conducted to investigate the effectiveness of rTMS in treating MDD in patients with atrial fibrillation. One study by Stultz et al. in 2022 studied the use of rTMS in a 55-year-old male with treatment-resistant depression and chronic atrial fibrillation. The patient had a cardiac pacemaker and an implantable defibrillator. In the study, they treated the patient using an H-1 coil over the left DLPFC at 18 Hz, 120% MT, 55 trains for 2 seconds with a 20-second intertrain interval for 1980 pulses per session. The patient’s post-treatment Beck Depression and PHQ-9 scales were 5 and 4, respectively (19). Thus, the study by Stulz et al. provides preliminary evidence of rTMS’s safety and compatibility in a patient with a pacemaker and a cardiac implant. However, our study is the first study to demonstrate safety and efficacy in treating comorbid MDD with GAD in a patient with AF. Furthermore, our study demonstrated the first use of a figure-8 coil over the left DLPFC at 10 Hz in a patient with MDD comorbid with AF.

Another critical factor to consider when treating patients with AF is the presence of autonomic dysfunction (20). In a meta-analysis by Lee et al., improvements in autonomic system control were observed when an excitatory rTMS protocol was applied to the left DLPFC. The study also showed significantly decreased blood pressure and heart rate following excitatory rTMS protocols. In addition, they found that an excitatory rTMS protocol showed significant positive effects on heart rate variability (HRV) variables (21). Future studies could examine the effects of rTMS on cardiovascular autonomic control in patients with AF using excitatory protocols targeting the left DLPFC, further reinforcing rTMS as a suitable therapeutic option.

## Limitations

The use of rTMS in treating recurrent MDD and GAD comorbid with AF was limited to a single case study. Additionally, the lack of long-term follow-up data prevents conclusions on sustained efficacy and safety. The efficacy and safety of rTMS in this population must be studied using large-scale, randomized, and blinded trials to validate the findings. Other limitations include potential response bias due to the use of self-reported psychometric assessments.

## Conclusion

We report the successful use of rTMS in achieving a significant reduction in MDD symptoms in our patient, who was comorbid with atrial fibrillation. However, this study provides only preliminary evidence, and the efficacy and safety of rTMS in this population must be evaluated in large-scale, randomized, blinded trials.

## Data Availability

The original contributions presented in the study are included in the article/supplementary material. Further inquiries can be directed to the corresponding author.
